# Radiofrequency wire ‘power wire’ recanalization of calcified chronically occluded inferior vena cava

**DOI:** 10.1186/s42155-018-0030-4

**Published:** 2018-11-22

**Authors:** Abhijit Salaskar, Michael Ferra, Harish Narayanan, Rishi Sood, Daniel Scher, Albert Chun, Anthony Venbrux, Shawn Sarin

**Affiliations:** Interventional Radiology Department, George Washington University Hospital, Washington, DC USA

**Keywords:** Inferior vena cava, IVC, Inferior vena cava occlusion, Inferior vena cava recanalization, Radiofrequency wire, Power wire

## Abstract

**Background:**

Radiofrequency (RF) wire recanalization of short segments of central venous obstruction has been considered safe; however its use for recanalization of long segments of inferior vena cava (IVC) has not been reported.

**Case presentation:**

A 55-year-old female with recurrent massive hematemesis was found to have systemic venous upper esophageal varices on endoscopy and an extensive chronic IVC occlusion on CT. Using both a percutaneous transhepatic and transfemoral approach IVC recanalization was performed. A snare was advanced to the cavo-atrial junction via transhepatic venous access. From the groin utilizing RF wire steerable guide sheaths, endovascular reconstruction of the IVC was performed. Post recanalization venography demonstrated patent stented IVC and marked decrease in the intraabdominal-pelvic collaterals. No recurrence of hematemesis was noted. After 6 months, patient remained asymptomatic and had functioning right femoral arteriovenous hemodialysis graft.

**Conclusions:**

Using appropriate techniques, Power wire recanalization of long occlusive segments of IVC can be safe and effective.

## Background

Recanalization of central venous obstruction with RF wire has been considered safe and effective based on outcomes of prior reports (Guimaraes et al., 2012). However in these studies, RF wire was used for recanalization of short segments of occlusions within subclavian vein (SV), brachiocephalic vein (BV) and superior vena cava (SVC) (Guimaraes et al., 2012). Easy penetrability of RF wire poses the risk of damaging virtually any soft tissues including critical arteries in the vicinity while crossing through long segments of occlusive organized scar tissues. Moreover advancing RF wire in caudal to cranial direction poses the constant risk of destabilizing RF wire tip due to motion of the heart. To our knowledge, RF wire recanalization of long segments of inferior vena cava (IVC) has not been reported. This case report is of a patient who had repeated episodes of life threatening hematemesis from upper esophageal varices secondary to chronic occlusion of the SVC and IVC. The IVC occlusion was successfully traversed with an RF wire and reconstructed with endovascular techniques after standard recanalization methods failed.

## Case presentation

**Fig. 1:**
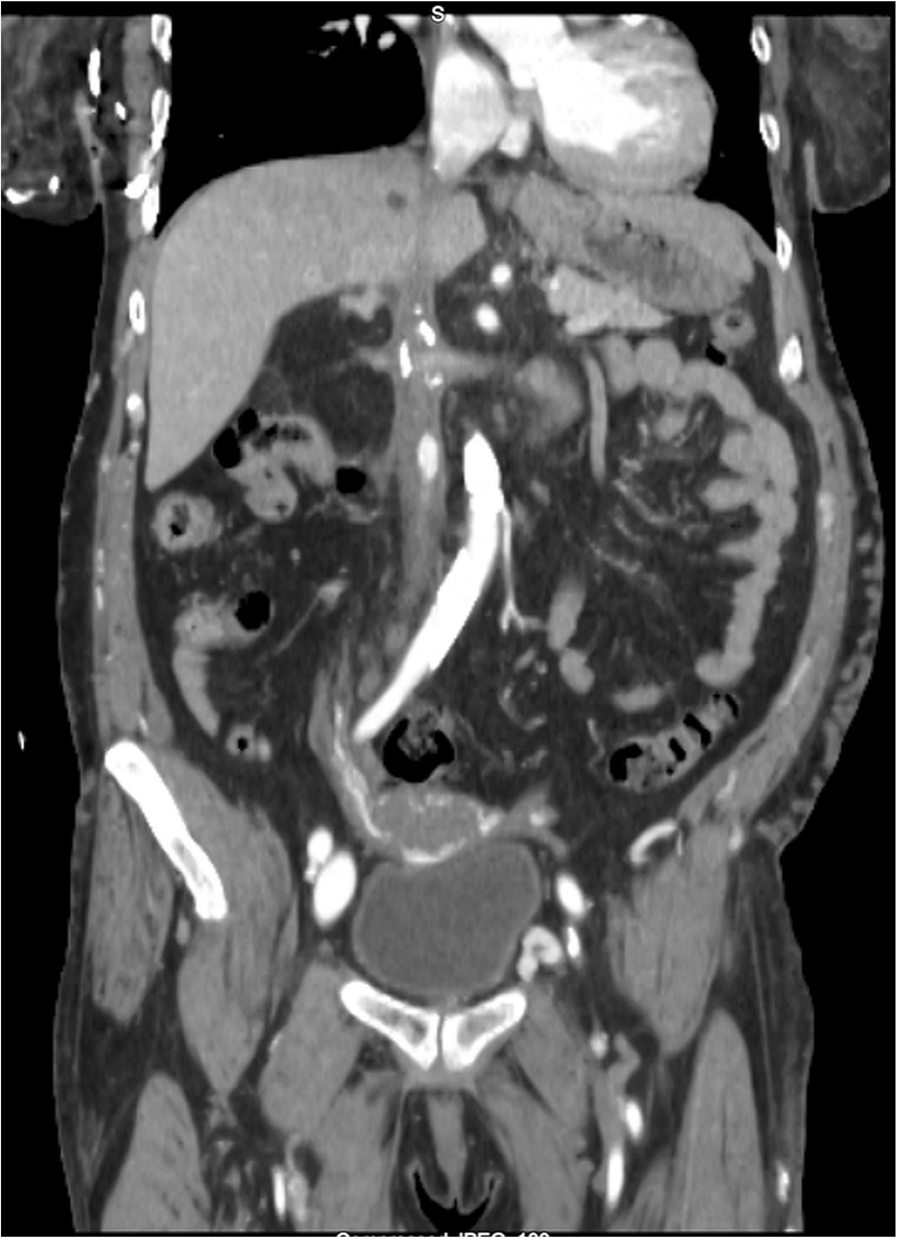
Initial CT scan showing calcified chronic occluded IVC and abdominal wall collaterals

**Fig. 2:**
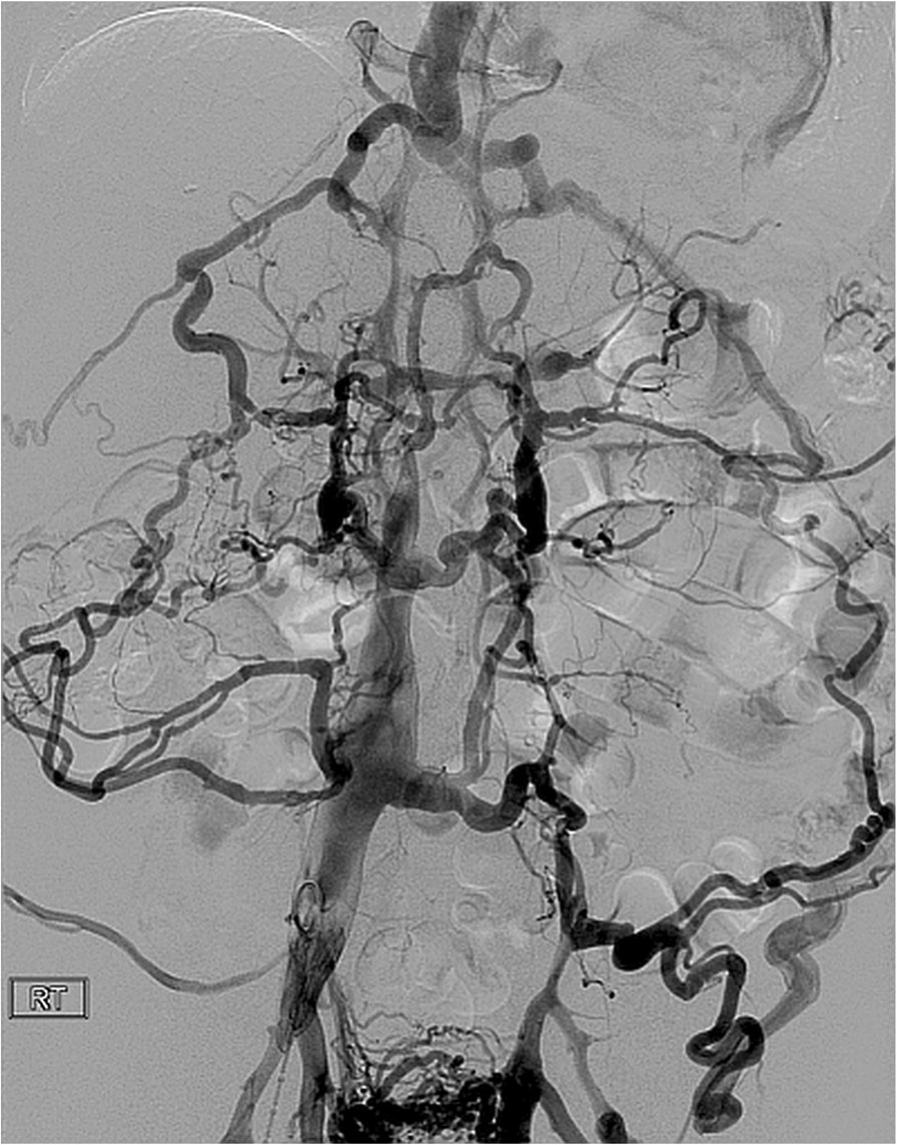
Pre-recanalization venogram showing completely occluded abdominal IVC and multiple abdominal-pelvic collaterals reconstituting flow through dilated azygos system

**Fig. 3:**
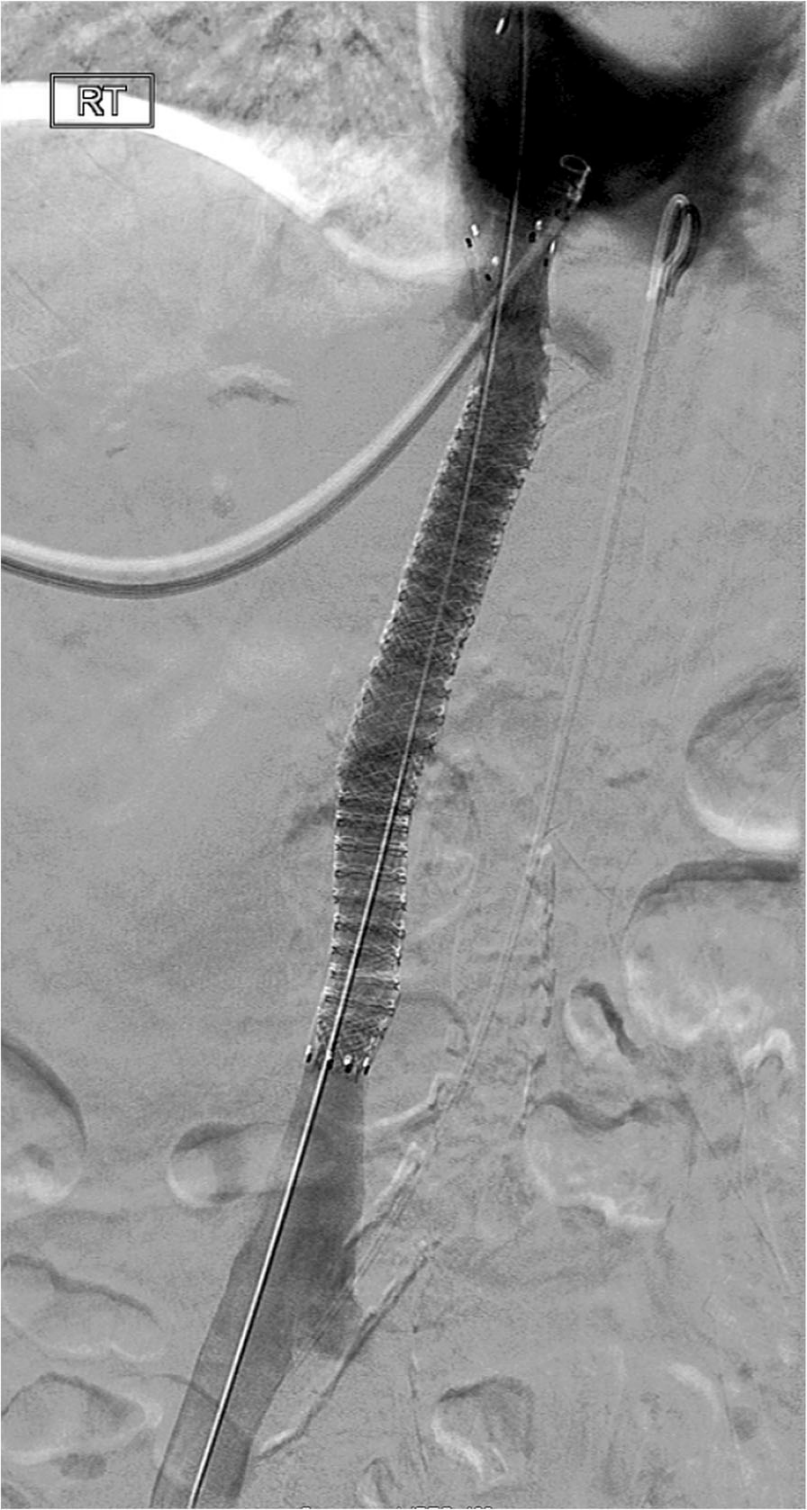
Immediate post-recanalization venogram showing widely patent stented IVC with decreased abdominopelvic collaterals, VCF-1 catheter in the aorta, transhepatic GooseNeck snare

The patient is a 55-yo-female with past medical history of end stage renal failure on hemodialysis with chronic SVC and IVC occlusions from prior hemodialysis accesses. She also had a history of chronic pancreatitis, coronary artery disease. She presented with recurrent massive episodes of hematemesis. Upper GI endoscopy 3showed varices only in the upper esophagus but none in the lower esophagus. The patient did not have liver disease. Cross sectional imaging at that time revealed extensive IVC occlusion (Fig. 1) and multiple dilated collaterals in the soft tissues especially around the area of the upper esophagus. Hence at the present hospital admission, after hemodynamically stabilizing the patient, interventional radiology (IR) was consulted for palliative central venous recanalization in hopes of reducing pressure in the aforementioned systemic venous collaterals. Since the patient also had a poorly functioning right groin hemodialysis arteriovenous (AV) graft with exhausted upper & lower extremity access sites and SVC obstruction, it was elected to recanalize the IVC first. It was hoped that this would not only decompress the esophageal varices through collaterals, but also improve function of the existing groin access. Multiple initial attempts to recanalize the occluded IVC using traditional techniques, including sharp recanalization with a variety of hydrophilic wires, catheters, triaxial systems and intravascular needles were unsuccessful. Initial planning venography from right common femoral access showed a completely occluded abdominal IVC and multiple abdominal-pelvic collaterals reconstituting flow through a dilated azygos system (Fig. 2). Five days later, patient was again brought to IR suite with an intention to recanalize occluded long segment of IVC with the use of RF wire ‘Power wire’ (Bayliss Medical). General anesthesia was administered. Under ultrasound guidance, 10 Fr and 5 Fr vascular sheaths were inserted into the right common femoral vein (CFV) and right common femoral artery (CFA) respectively. Then 4 Fr VCF-1 flush catheter was positioned in the abdominal aorta through CFA sheath to serve as a marker for the abdominal aorta. Venography redemonstrated abdominal IVC occlusion starting from confluence of the common iliac veins to intrahepatic IVC, innumerable abdominal-pelvic collaterals and dilated azygos/ hemiazygous system. Though not well demonstrated on cross sectional imaging or venography, it was assumed that the cavo-atrial junction could be patent and it could be accessed via transhepatic venous access. Using percutaneous transhepatic approach, a 23 cm long 6 Fr vascular sheath was placed in the proximal part of the right hepatic vein. Hepatic venogram confirmed the patency of all three hepatic veins and patency of a short segment of the cavo-atrial junction. Through intrahepatic vascular sheath, a 25 mm GooseNeck snare was positioned at the cavo-atrial junction. The snare served as a target for recanalization procedure. Following this, a 7 Fr steerable sheath was inserted through the right CFV sheath. Through steerable sheath, a co-axial system of catheters was assembled to guide the RF wire towards the snare in the cavo-atrial junction. The length of the occlusion was estimated to be greater than 10 cm. With great care, the RF wire and the coaxial system were advanced at 1–2 mm increments, always noting the relation of the RF wire to the aortic flush catheter in multiple oblique images. The RF wire generator was set to create short 300 milliseconds duration RF delivery. When the Power wire was approaching the proximal part of the intrahepatic IVC obstruction, the 25 mm snare was replaced with a 5 mm snare for more precise targeting. The 5 mm snare was positioned through an IMA catheter advanced through the intrahepatic vascular sheath so that it could be directed towards the approaching RF wire. The 5 mm snare captured the RF wire. Both the snare and RF wire were advanced into the right atrium. At this point IVC obstruction had been traversed. The RF wire was exchanged for a Lunderquist wire and catheters were removed. The IVC occlusion was dilated with 4 mm × 200 mm and 8 mm × 200 mm Mustang balloons. Because of the length of the IVC occlusion traversal, and potential for IVC injury, it was elected to use covered stent grafts except at the confluence of the hepatic veins. It was thought that bare metal self expanding stent across the origins of hepatic veins would reduce the risk of hepatic venous thrombosis. Hence initially a 14 mm × 120 mm bare metal self expanding E. Luminexx stent was deployed. Within this stent, three Gore Viabahn balloon expandable stent grafts (5 mm × 39 mm, 5 mm × 59 mm, 7 mm × 39 mm) were deployed except at the confluence of the hepatic veins. Acknowledging that the atretic renal veins would be covered it was decided to use stent grafts to seal any potential vascular injury. All the stents were then post dilated with a 14 mm × 40 mm balloon. Repeat venography demonstrated brisk flow through the recanalized IVC and a marked decrease in the number and sizes of intra-abdominal pelvic collaterals (Fig. 3). A 28 cm hemodialysis catheter was placed through the right hepatic vein access to obtain hemostasis at this access. Hemostasis at other groin access sites were obtained with manual pressure. There were no hematomas and pedal pulses remained unchanged. The patient was observed in the intensive care unit overnight.

**Fig. 4:**
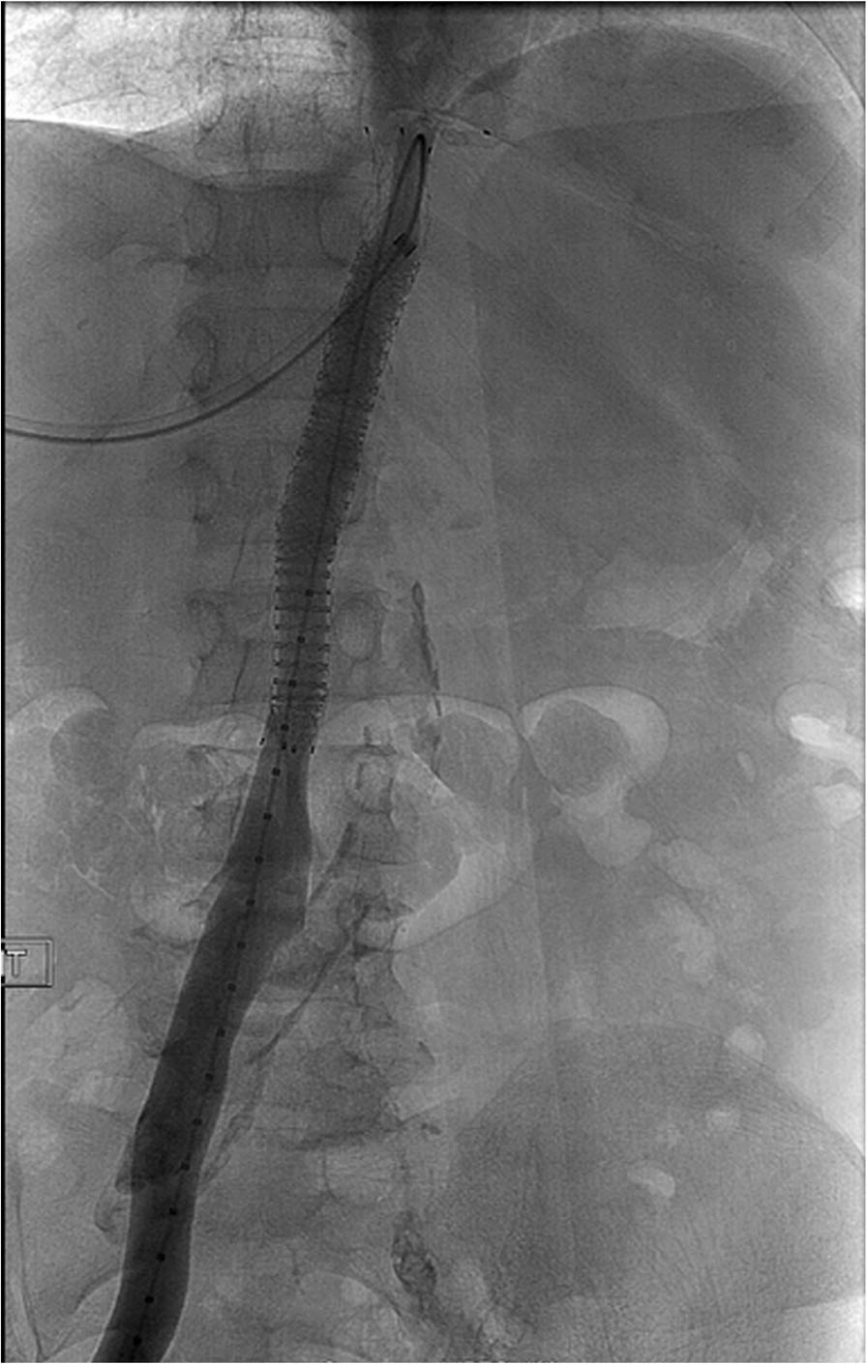
One month follow up venogram showing widely patent IVC stents. Please note the sheath in the hepatic vein prior to deploying a vascular plug

One month later, the patient returned for follow up venography, which demonstrated widely patent stented IVC and removal of the transhepatic permcath (Fig. 4). Nephrology service noted improved hemodialysis and significant improvement in patient’s volume status. Hematemesis did not recur. Six months post-procedure, patient remained asymptomatic and had functioning right femoral arteriovenous hemodialysis graft.

## Conclusions

RF wire recanalization has been successfully used to treat pulmonary atresia, to create atrial septal perforation, to recanalize completely occluded thoracic aorta and to recanalize chronic or malignant central venous obstructions after failure of conventional endovascular techniques (Guimaraes et al., 2012; Auyang et al., 2018). In these studies, RF wire successfully recanalized upto 10 cm long chronic occlusions within SV, BV and SVC (Guimaraes et al., 2012). However use of RF wire to cross long IVC occlusions has not been reported. Anticipated obstacles for RF wire recanalization were long course of IVC, tough organized scar tissue within these chronic occlusions, risk of damaging critical neighboring structures due to high penetrability and flexibility of RF wire tip, and constant destabilization of RF wire tip due to motion of the heart.

In order to safeguard neighboring critical structures, we used following techniques. We placed indicator VCF-1 catheter in the aorta and constantly monitored Power wire tip to avoid deviation towards periaortic area. Unpredictable movement of the RF wire tip was a major concern while crossing long occlusive segment in caudal to cranial direction, especially when the flexible tip moves away from the power generator towards the beating heart. Use of steerable sheath allowed us to have better control of RF wire tip. Also by placing a 25 mm snare tip at the IVC-atrial junction, Power wire trajectory was given a precise target.

Due to easy penetrability of heated tip of RF wire, prior studies used techniques such as short duration of RF energy delivery and very small advancements of RF wire tip (Guimaraes et al., 2012). We used 300 milliseconds duration RF delivery pulse and traversed only 2 mm during each pulse. After 2 mm cranial advancement, multiple real time anteroposterior (AP) & oblique views were obtained to confirm the intraluminal location of RF wire. Use of cone beam CT could be an additional safe alternative to monitor the RF wire trajectory (Auyang et al., 2018).

Multiple cranial and/or femoral access approaches have been reported in the literature to treat chronic complex central venous occlusions (Massmann et al., 2016). We chose combined femoral and transhepatic approach due to simultaneous presence of SVC obstruction. Transhepatically placed snare not only helped us to direct the trajectory of RF wire but also to ensnare tip of the RF wire. As the wire crossed the intrahepatic IVC obstruction, we snared the tip of the wire and then advanced the IMA catheter containing goose neck snare into the right atrium. This maneuver allowed the safe and predictable passage of snared Power wire from intrahepatic IVC to right atrium, in the vicinity of beating heart. Thus with the use of appropriate techniques; Power wire recanalization of long occlusive segment of IVC has shown to be safe and effective.

## Abbreviations

AP: Anteroposterior
AV: Arteriovenous
BV: Brachiocephalic vein
CFA: Common femoral artery
CFV: Common femoral vein
IR: Interventional radiology
IVC: Inferior vena cava
RF: Radiofrequency
SV: Subclavian vein
SVC: Superior vena cava
