# Correction to: Radiofrequency wire ‘power wire’ recanalization of calcified chronically occluded inferior vena cava

**DOI:** 10.1186/s42155-019-0082-0

**Published:** 2019-12-19

**Authors:** Abhijit Salaskar, Michael Ferra, Harish Narayanan, Rishi Sood, Daniel Scher, Albert Chun, Anthony Venbrux, Shawn Sarin

**Affiliations:** Department of Interventional Radiology, George Washington University Hospital, Washington, DC, USA

**Correction to: CVIR Endovasc**

**https://doi.org/10.1186/s42155-018-0030-4**

In the published article (Salaskar et al., 2018) the statement under the subheading ‘Consent for publication’ is incorrect.

“IRB approval not needed for this type of submission at our institution.”

should read:

“Informed consent for publication of this case report and its accompanying images has been obtained from the patient”.
